# Sensitive nerve function measurement in facial trauma: An observational study

**DOI:** 10.4317/jced.56830

**Published:** 2021-01-01

**Authors:** Edson-Luiz Cetira-Filho, Fábio-Wildson-Gurgel Costa, Saulo-Ellery Santos, Manoel-de Jesus-Rodrigues Mello, Paulo-Goberlânio-de Barros Silva, Andréa-Silvia-Walter de Aguiar

**Affiliations:** 1 DDS,OMS, MSc student. Oral and Maxillofacial Surgeon and master’s in science student in Federal University of Ceará (UFC). Professor of Mauricio de Nassau University (UNINASSAU), Fortaleza, Ceará, Brazil; 2 DDS, OMS, MSc, PhD. Oral and Maxillofacial Surgeon, MSc, PhD, Associate Professor, Department of Clinical Dentistry, UFC, Fortaleza, Ceará, Brazil; 3 DDS, OMS, MSc, PhD. Oral and Maxillofacial Surgeon, MSc, PhD, Associate Professor, Division of Oral Surgery, UNIFOR, Fortaleza, Ceará, Brazil; 4 DDS, OMS, MSc, PhD. Oral and Maxillofacial Surgeon, MSc, PhD. Professor of UNICHRISTUS, Fortaleza, Ceará, Brazil; 5 DDS, Oral Pathologist, MSc, PhD. Professor of UNICHRISTUS, Fortaleza, Ceará, Brazil; 6 DDS, Oral and Maxillofacial Surgeon, MSc, PhD, Associate Professor, Department of Clinical Dentistry, UFC, Fortaleza, Ceará, Brazil

## Abstract

**Background:**

Facial trauma is responsible for various types of health damage and may be functional or aesthetic. Depending on the degree of energy released in this type of trauma, sometimes an irreversibility degree is obtained. This study aimed to perform an objective evaluation of traumatic peripheral nerve injuries resulting from mandibular fractures and midface, using silicon monofilaments.

**Material and Methods:**

This was an observational, cross-sectional study. All patients with maxillofacial fractures, who were hospitalized by the department of Oral and Maxillofacial Surgery of Instituto Dr. José Frota Hospital, were randomly recruited and screened for inclusion in the present study. Sixty patients, victims of automobile accidents or firearms, were evaluated using Semmes Weinstein monofilaments in the regions corresponding to the mental and infraorbital nerves, right and left.

**Results:**

The highest frequencies mandibular nerve changes were those that there was a loss protective sensation, but in which, the patient can feel deep pressure and pain; In which the worst sensory alterations occurred in patients’ victims of firearm. In the middle third of the face, the worst alterations were those that there was a loss of the protective and discriminating sensation for hot and cold.

**Conclusions:**

The use of monofilaments is a support tool in oral and maxillofacial traumatology for the diagnosis and monitoring of peripheral sensory alterations.

** Key words:**Peripheral nerve injuries, facial trauma, wounds and injuries, accidents, traffic, violence.

## Introduction

The facial fractures occur in a significant proportion worldwide, and epidemiology varies according to the population, in which accidents with motor vehicles, physical aggressions, and falls are the main etiological agents (1-4). The mandible and maxillary bones are the most affected (4). Signs and symptoms presented after facial fractures include the nerve lesions of the inferior alveolar nerve and its terminal ramifications - mental nerve, in cases of mandible fractures (5); or infraorbital nerve, in cases of fractures of the middle third of the face (6), either in the maxillary and/or zygomatic bones. The consequences of nerve lesions may range from paresthesia to dysesthesia, in which the first is defined as partial loss of sensation, but with some sensation of touch; While the second is the partial loss of sensation accompanied by a painful component or discomfort (7).

In order to establish an early diagnosis of sensory lesions, to determine their extent, duration and/or regression, emphasizing the objective methods with the use of silicone monofilaments. The most widespread tests are those that use static two-point discrimination and using silicon/nylon monofilaments (8), the Semmes Weinstein® esthesiometer (Sorri, Bauru-SP, Brazil) (9).

In Oral and maxillofacial surgery, these monofilaments have been applied in studies of mandibular sensory alterations after third molars extractions, as well as after sagittal osteotomies in orthognathic surgery, due to the probability of injury to the inferior or mental alveolar nerve (10,11).

Up to the present, there was identified a publication that the esthesiometers were used in the evaluation of sensorial changes of the middle third of the face, after a traumatic event, which makes this proposal a vanguard (12).

This work aimed to evaluate of sensory alterations of the mental and infraorbital nerves in patients with facial trauma in a public reference trauma hospital, using Semmes Weinstein® esthesiometer (Sorri, Bauru-SP, Brazil).

## Material and Methods

The research protocol was submitted to the Ethics Committee in Human Research at Instituto Dr. José Frota Hospital, having been approved and licensed under the protocol 1.439.895 on 01/02/2016. The Helsinki Declaration was read and followed its guidelines in this investigation.

This was an observational and cross-sectional study. This study followed the STROBE protocol. All patients with maxillofacial fractures, who were hospitalized by the department of Oral and Maxillofacial Surgery of Instituto Dr. José Frota Hospital, random recruitment was screened for inclusion in the present study. Patients hospitalized in critical units (Intensive Care Unit, Burn Treatment Center, Anesthetic Recovery Room, Risks I and II), as well as patients with panfacial fractures, patients medicated with depression of the central or peripheral nervous system and/or with cognitive or sensorial deficit were excluded. An estimated 23 patients were expected to be hospitalized during a month, and the length of this study was from March to June of 2016. Based on a sample size estimation corresponding to 95% confidence interval (CI), the final sample was 60 patients. The patients participating in this study were thoroughly informed of the advantages and disadvantages of this research, and their consent was documented.

An evaluation of the sensorial alterations was performed in 60 patients, of both sex, victims of traffic accident (TA) and interpersonal physical violence (IPV), who were assisted in the oral and maxillofacial surgery department. The sensory evaluation was performed using the Semmes Weinstein® esthesiometer (Sorri, Bauru-SP, Brazil). The purpose of this work was to evaluate and quantify the pressure threshold in mental and infraorbital nerve-related dermatomes of the facial skin in patients affected by facial trauma. The calibration of each filament is provided by the manufacturer and ranges from 0.05 to 300 gf. This test involves five monofilaments that are scored in terms of milligrams (mg) (green (0.05 mg), blue (0.2 mg), violet (2.0 mg), dark red (4.0 mg) and magenta (300 mg)), beginning with a baseline value of 0.05 mg, which indicates a “normal sensation”.

**Figure 1:**
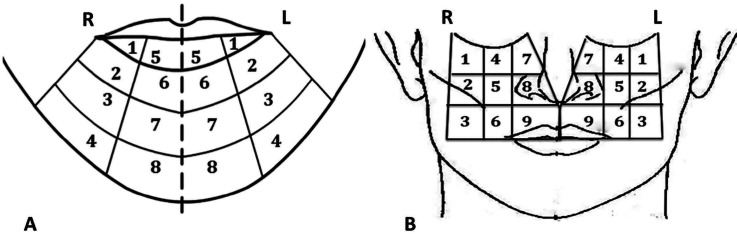
Distribution of the sensory evaluation zones in the mandibular region (A) and the middle third of the face (B).

The facial areas were those corresponding to the innervation of the right and left mental and infraorbital nerves. In order to evaluate the inferior alveolar nerve injuries were chose the demarcation proposed by Nguyen *et al.* (13) in the bilateral mental region. Due to the lack of demarcation for the region innervated by the infraorbital nerves, the authors of this article propose a division of the region corresponding to the innervation, right and left, also for the same purposes (Fig. 1).

The tactile evaluation was performed according to the manufacturer and each region classified according to the strength and explained in scores ranging from (1) - without evident change to (7) - absence of sensitivity (14).

Standardized testing protocol

The filament needs to be applied perpendicular to the test site, and the pressure was slowly increased until the filament begins to bend. Only one reviewer performed all assessments. The protocol was performed using each filament for 30 seconds if it did not bend, with continuous use of manual force by the examiner.

The patient evaluation was performed before the surgical intervention, in areas without wounds or lacerations, when it was clinically observed an edema reduction after the trauma. The face sides of all patients were evaluated; in those whom had trauma or bone fracture on only one side of the face, the other side served as a control group, being split-face.

In addition, patients who used alcohol or narcotics were evaluated after clinical reestablishment from the psychological point of view and were no longer under the effects of these compounds.

Calculation of sensory loss

The esthesiometric evaluation was tabulated as increasing scores of sensory loss, with 1 = green; 2 = blue; 3 = purple; 4 = red; 5 = orange; 6 = pink; 7 = black), with the sensory loss ranging from 1 (absent sensory loss) to 7 (maximum sensory loss).

After that, the mean of the sensory loss scores between the right and left sides was calculated. For the individuals in whom the assessment was made only on one side, only that side was considered for calculation of the mean. Likewise, the mean of the sensory loss between the maxilla and the mandible was calculated. For individuals in whom the evaluation was made only in a jaw, or for sites only in the maxilla (anatomic site 9), only that site was considered for calculating the mean. Thus, the mean of the sensory loss score ranged from 1 and 7.

The average sensory loss was adjusted on a scale from 0% to 100% (Percentage Sensitive Loss, PSL), with 1 corresponding to 0% and 7 corresponding to 100%: PSL = mean of the sensory loss score * 100 / 1).

-Statistical analysis

The medians and minimum and maximum values, as well as the mean and standard deviation of the sensory loss scores assessed by the esthesiometry and the PSL calculation, were calculated. Multiple linear regression model was used to evaluate the weight that each region had in the PSL calculation. Additionally, the patients were categorized as low sensorial loss and high sensorial loss based on the median PSL and were crossed with the other sociodemographic and clinical-surgical variables to evaluate risk factors for sensorial loss after injury to the maxillofacial region. The variables that showed *p* <0.200 were inserted in a multinomial logistic regression model.

## Results

-Clinical and surgical characterization

The sample consisted of 60 individuals, of which 38 (63.3%) suffered automobile accidents and 22 (36.7%) were victims of violence. The middle third region was reached in 32 (53.3%) patients and the mandible in 28 (46.7%). The majority were male (n = 48, 80.0%), were over 25 years old (n = 31, 51.7%), single (n = 49, 81.7%), with elementary School incompleteness (n = 35, 58.3%), dark skinned (n = 31, 51.7%), were informally employed (n = 20, 35.7%) and lived in their own homes (n = 37, 62.7% (Table 1).

**Table 1:**
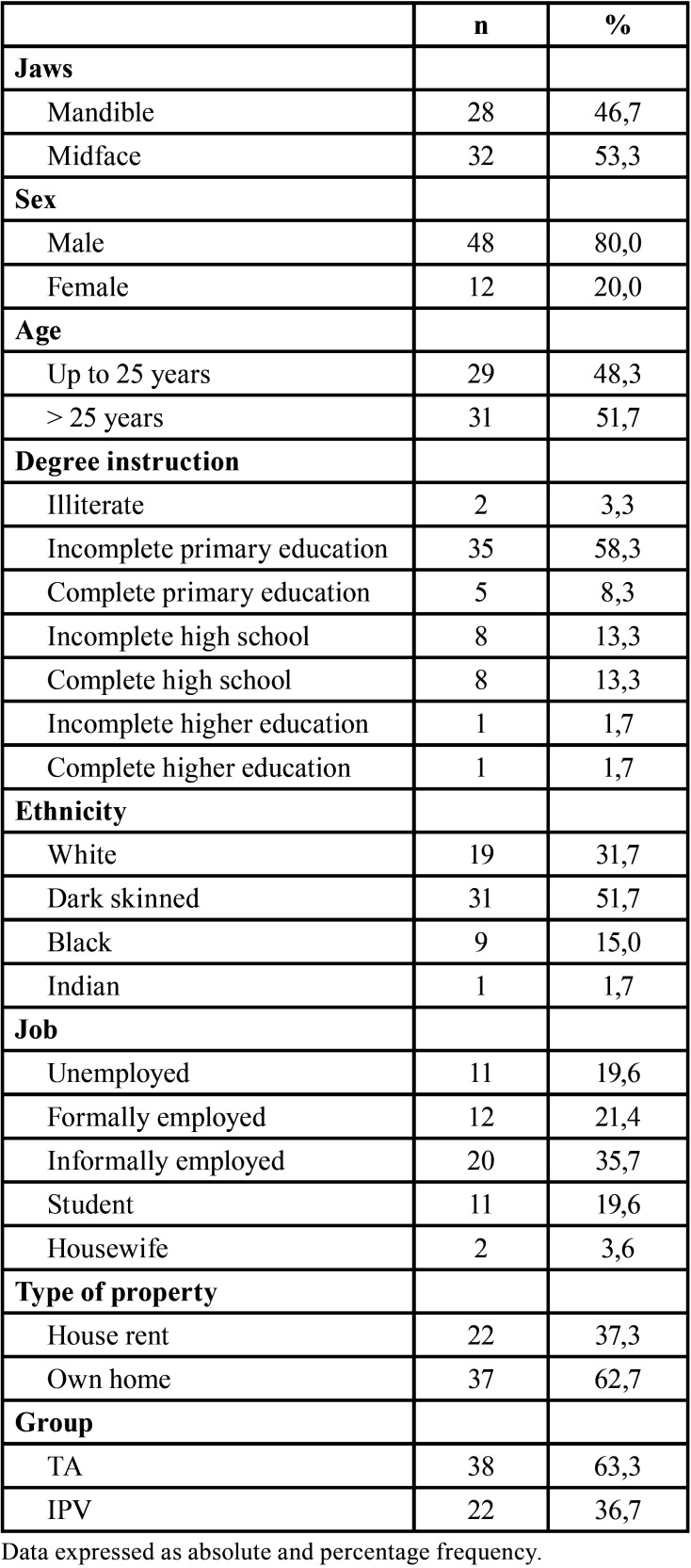
Sociodemographic characterization of patients with maxillofacial injury submitted to an esthesiometry analysis.

Alcohol consumption was reported in most patients (n = 43, 71.7%), as well as narcotic (n = 55, 91.7%). Most patients reached the unconscious service (n = 32, 53.3%) and had the affected right side of face (n = 21, 39.6%), traces of fractures 1 (simple) (n = 26, 49.1%). Approximately one-third of the sample was not hospitalized, while about one-third were hospitalized for up to 14 days and the remainder were hospitalized for more than 14 days. Of the 35 patients operated on, most used titanium plates 1.5 (n = 17, 48.6%) and more than one plaque (n = 23, 65.7%) (Table 2).

**Table 2:**
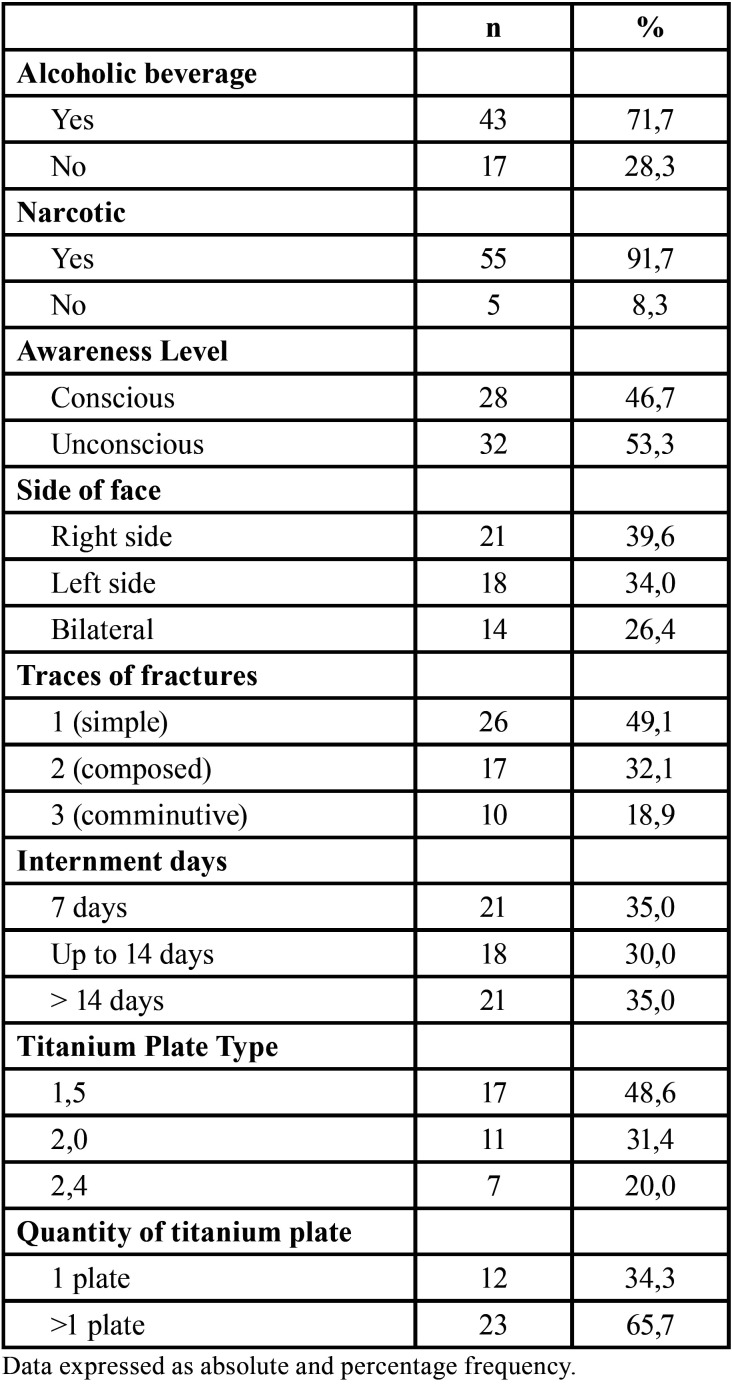
Characterization of risk factors and clinical-surgical variables of patients with maxillofacial injury submitted to an esthesiometry analysis.

Of the patients who received a majority (n = 15, 68.2%) were by firearm and seven cases (31.8%) were associated with beatings. The most common etiological agent was perforating (n = 12, 57.1%), followed by the blunt (n = 6, 28.6%) and perforating (n = 3, 14.3%). Firearm projectile was observed in 13 (86.7%) patients.

Motor vehicle crashes or collision between motor vehicles or between motor vehicle and fixed bulkhead were the etiological factor of 14 patients each (36.8%). Overwhelming was observed in 5 patients (13.2%) and other types of accidents including collision between motorcycle and bicycle were another five cases (13.2%). Motorcycles (n = 31, 81.6%) were the most used vehicle type, most were drivers (n = 20, 62.5%) and the others (n = 12, 37.5%) were passengers and helmet were used by most drivers (n = 14, 70.0%).

-Sensory loss analysis

The largest medians of sensory loss on the right side were observed in areas seven to nine and on the left side in areas four, five and seven to nine. The highest mean between the two sides was observed in areas seven to nine, with the patients presenting an average of 14.52 ± 18.50% of sensory loss in the assessed region, the median being 8.33% ranging from 0.00% to 91.67%. The mean value between the right and left sides of all the evaluated regions contributed significantly to the PSL calculation, being the smallest contribution of area 3 (7.9%) and higher contribution of areas 4 (15.5%) and 7 (13. 4%). Evaluating by face segments, the mandible had the worst averages in areas 5 (1.00±1.54), 6 (1.00±1.54), and 7 (1.02±1.54). While in the middle third, the worst averages were in areas 7 (0.70±0.69) and 8 (0.70±0.71), the area closest to the exit of the mental foramen and the infraorbital foramen respectively (Table 3).

**Table 3:**
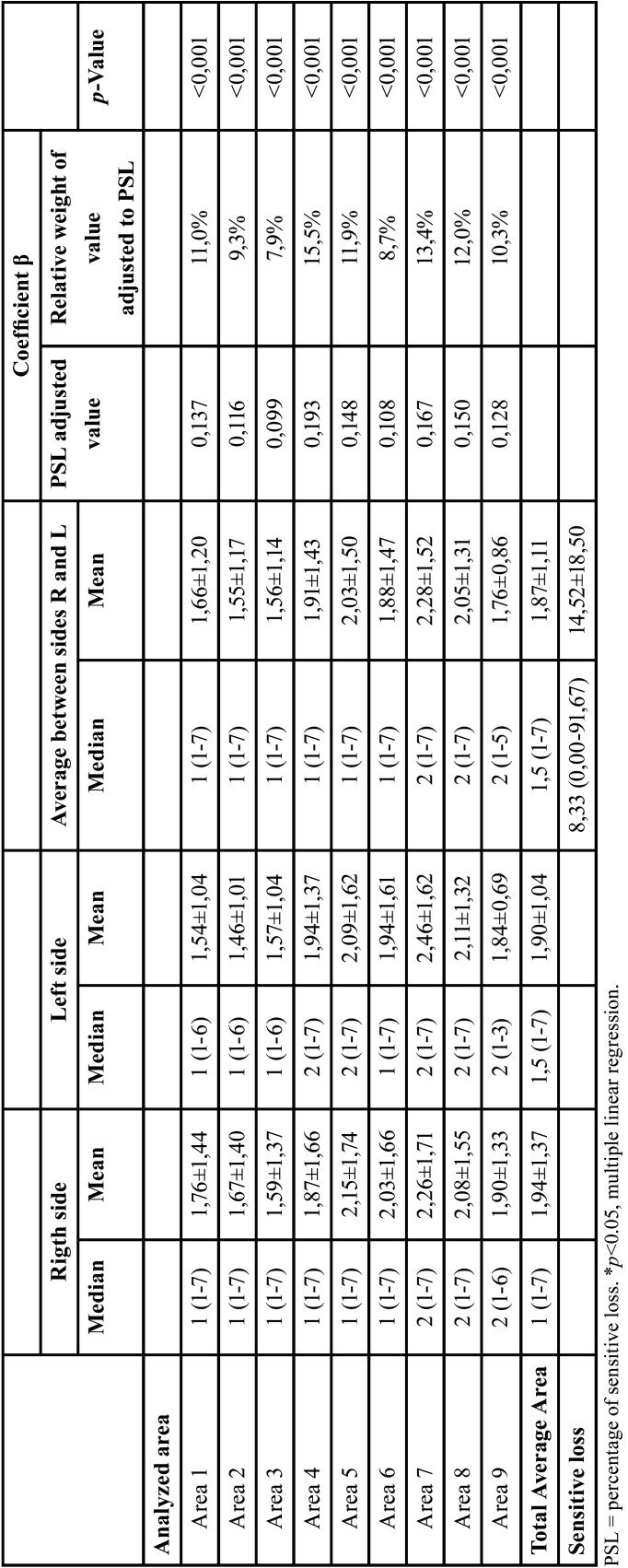
Characterization of the sensory loss in patients with injury in the maxillofacial region submitted to an esthesiometry analysis.

Patients who experienced trauma due to violence (*p* = 0.047) and who had illiterate/primary education level (*p* = 0.009) were significantly more associated with sensorial loss than 8%, and this low level of education an independent factor that increased the prevalence of sensorial loss in the studied sample (Table 4) by 3.77 (95% CI = 1.04 - 13.66).

**Table 4:**
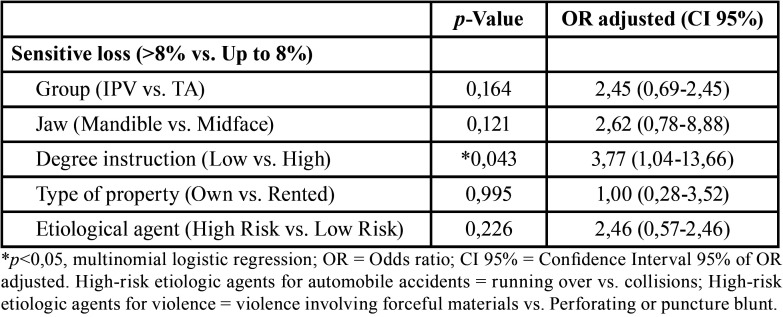
Main outcomes associated with PSL of patients with maxillofacial injury submitted to an esthesiometry analysis.

**Figure 2:**
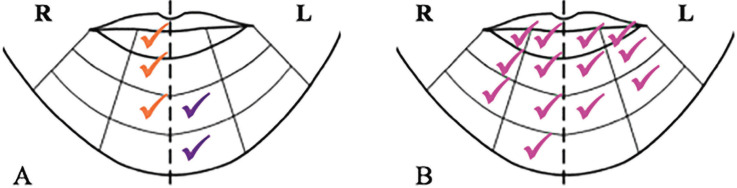
General distribution of the worst alteration of sensibility in the mental region due to auto accident and interpersonal physical violence. Colors: Orange – Loss of protective sensitivity; Able to feel deep pressure and pain. Violet – reduced protective sensitivity, light touch remaining enough to prevent injury; Difficulty with form and heat discrimination and Magenta - loss of sensitivity to deep pressure.

For the neurological changes in the mandible, it was observed that victims of TA had sensorial alterations corresponded to the orange filaments - in which there is loss of the protective sensation, in 23.1%; and violet - with decreased protective sensitivity, in 30.8%. However, in both, there is a permanence of sensation of deep pressure and pain. The worst results of sensory alterations occurred in patients that were victims of IPV, which pink or magenta filaments, corresponding to loss of texture discrimination (mild touch), were related in 42.9% as well as inability to discriminate forms and temperature, were the most observed (Fig. 2).

**Figure 3:**
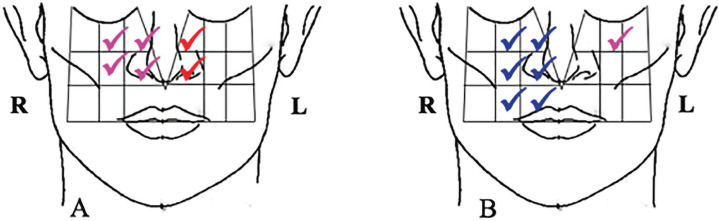
Distribution of the loss of sensitivity to deep pressure, not being able to feel pain in the mental region due to auto accident and interpersonal physical violence.

Nevertheless, in both TA (14.3%) and IPV (7.7%) victims, there was loss of sensitivity to deep pressure, in which the individual does not normally feel pain, in which the manufacturer points out as areas of paresthesia (Fig. 3).

Regarding the nerve alterations of the middle third of the face, it was observed that the worst sensory alterations occurred in the areas near the nasal region. In the TA patients, loss of sensitivity to deep pressure (pink/magenta filament) was found in 16.7% and the loss of protective and discriminatory sensation for hot and cold (red filament) was the most frequent in 12.6% patients. On the other hand, in the cases of IPV, the most frequent sensorial alteration was the decrease of sensitivity with difficulty to the fine discrimination (blue filament) – 12.5%. For the middle third of the face, there was no loss of sensitivity to deep pressure.

## Discussion

This investigation highlighted the distribution of fractures, the occurrence ratio of different types of fractures, the traumatic reasons, and the extent of sensitive nerve injury.

The facial anatomy is the core that guides the professional regarding the diagnosis and treatment of the patient victim of facial trauma. Mandibular sensorial alterations may be due in fractures that involve the inferior alveolar nerve or its branch, the mental nerve. The latter provides sensitivity to the lower lip and labial mucosa, the canine teeth and lower premolars (15).

According to Burns *et al.* (16), mandibular fractures can generate sensory alterations, whose clinical repercussions may be in the area of the mental nerve, promoting damage to patients. Similarly, lesions in the proximal maxillary bones or in the region of the infraorbital foramen, in which the infraorbital nerve emerges, lead to sensory alterations in the lower palpebral region, nose wing and upper lip (17).

The monitoring of sensory changes brings benefits to patients, both in the early diagnosis of the change and in the clinical follow-up of the lesion progression or regression (18).

For the sensorineural evaluation of the facial traumatized patient, a range of mechanoreceptive and nociceptive tests, based on the specific receptor stimulus, can be used. The static light touch test, for example, evaluates the perception of pressure through Von Frey monofilaments.

The use of the Semmes Weinstein® esthesiometer, for the evaluation of sensorial alterations, is diversified (19,20). There are studies that validated them as a screening tool for diabetic peripheral neuropathy (21), evaluation of carpal tunnel syndromes (20) and sensibility and evaluation after mammoplasty (22).

Although the Semmes Weinstein® esthesiometers (Sorri, Bauru-SP, Brazil) is not familiar to many oral and maxillofacial surgeons, it should be considered a relevant tool in sensorineural evaluation because it is an objective, easy to perform, reproducible and low-cost method. The monitoring of sensorial changes brings benefits to the patients, both in the early diagnosis of the change and in the clinical follow-up of the progression or regression of the lesion.

The mechanism is applied perpendicular to the skin and pressure is applied only until the filament begins to bend in sequential order and the patient senses that or not.

In the use of this objective method, many patients perceived the green filament stimulus, which demonstrates a degree of normality. Of those that presented diminished sensitivity, the blue, violet, magenta, black, orange and red filaments are highlighted in frequency of appearance.

It was observed that the worst mandibular sensorial manifestations were due to IPV, in which the magnitude of the trauma from firearms was, for the most part, a decisive factor for this (23).

The facial traumas can generate significant alterations to the patient’s victims of these types of accidents, as sensorial alterations, varying in degree. The use of monofilaments of Semmes Weinstein® can be a support tool in oral and maxillofacial traumatology regarding the early diagnosis of sensory alterations, having the advantages of being low cost and easy to use. In this way, the use of monofilaments in the examinations of patients with facial traumatisms is proposed, acting in the monitoring of such alterations and the clinical follow-up of the regression or progression of the nerve injury.
